# Individual and Interactive Effects of Adverse Childhood Experiences and Household Income on Adolescent Depression and Anxiety Symptoms

**DOI:** 10.1002/nur.70107

**Published:** 2026-07-18

**Authors:** Chelsea R. Moore, Philip T. Veliz, Alison L. Miller, Natasha V. Pilkauskas, Todd I. Herrenkohl, Sarah A. Stoddard

**Affiliations:** ^1^ School of Nursing University of Michigan Ann Arbor Michigan USA; ^2^ Division of General Pediatrics & Adolescent Health, Department of Pediatrics University of Minnesota School of Medicine Minneapolis Minnesota USA; ^3^ Health Behavior and Health Equity, School of Public Health University of Michigan Ann Arbor Michigan USA; ^4^ Gerald R. Ford School of Public Policy University of Michigan Ann Arbor Michigan USA; ^5^ School of Social Work University of Michigan Ann Arbor Michigan USA

**Keywords:** adolescent, adverse childhood experiences, child development, mental health, social determinants of health, socioeconomic factors

## Abstract

Cumulative Adverse Childhood Experiences (ACEs) and low household income are associated with adolescent depression and anxiety, but it is unclear whether distinct ACE subdomains – maltreatment (e.g., abuse) versus parent challenges (e.g., parental mental illness) – when experienced in distinct developmental periods exert a unique influence on adolescent mental health, or how ACEs and economic disadvantage operate together to influence outcomes. Thus, we aimed to (1) examine associations between baseline household income, early childhood ACEs (ages 0–5; sub‐indexed as Maltreatment vs. Parent Challenges) and adolescent depression and anxiety symptoms (age 15), and (2) investigate whether household income moderates the associations between ACE sub‐indices and each mental health outcome. We used serial linear regressions and data from the United States‐based, longitudinal, racially/ethnically and socioeconomically diverse Future of Families and Child Wellbeing Study (*n* = 2523; 51% female; 51% non‐Hispanic Black; 36% in poverty at birth). The Parent Challenges sub‐index (but not the Maltreatment sub‐index) was associated with depression and anxiety symptoms. Low household income was associated with depression symptoms. Income moderated the association between the Parent Challenges sub‐index and anxiety symptoms; youth from the lowest and highest income strata were at greatest risk. Strategies are urgently needed for ACE prevention and to support parents of very young children (or expectant parents) who are experiencing behavioral health challenges, interpersonal violence, criminalized behavior, and/or poverty to promote healthy child development and adolescent mental health. Health professionals are well‐positioned to advocate for supportive policies, promote trauma‐informed environments, and identify/address significant parental challenges early in development.

## 1 Introduction

Adolescent depression and anxiety are prevalent and associated with far‐reaching negative sequelae (Centers for Disease Control and Prevention 2024; Clayborne et al. 2019; Swan and Kendall 2016). Cumulative Adverse Childhood Experiences (ACEs; including abuse, neglect, and significant household challenges) are risk factors for depression and anxiety (CDC 2021; Petruccelli et al. 2019). However, most research on cumulative ACEs has operationalized ACEs with composite indices of different ACE types experienced before age 18 (Fitzgerald and Gallus 2024). This approach discounts the possibility that distinct ACE subtypes (e.g., maltreatment vs. parent challenges) may exert a unique influence on outcomes and the importance of ACE timing during development.

Childhood economic disadvantage is also associated with adolescent depression and anxiety through several pathways that span ecological levels, including factors within the macrosystem (e.g., decreased access to mental health services; Yoshikawa et al. 2012). Factors within higher‐order ecological levels are hypothesized to shape, or moderate, ACE effects on mental health (e.g., access to mental health care may mitigate the mental health sequelae of ACEs). However, such moderating effects are understudied. Thus, the purpose of this study was to (1) examine associations between *early childhood* ACEs (sub‐indexed as Maltreatment vs. Parent Challenges), household income, and adolescent depression and anxiety symptoms, and (2) investigate if household income moderates the associations between ACEs and depression/anxiety symptoms.

## 2 Background

### 2.1 Adolescent Mental Health

Adolescent depression and anxiety are prevalent in the United States (U.S.; Centers for Disease Control and Prevention (2019); Centers for Disease Control and Prevention 2024) and can substantially affect one's ability to function in daily life. Estimates suggest approximately 17% and 21% of adolescents experienced depression and anxiety symptoms, respectively, within the past 2 weeks (Centers for Disease Control and Prevention 2019); Centers for Disease Control and Prevention 2024). Moreover, an individual experiencing one condition is at increased risk of developing the alternative condition (Jacobson and Newman 2017). Adolescence is a sensitive developmental period where individuals establish their identities, autonomy, and increasingly important peer relationships (World Health Organization 2024). Depression and anxiety may impede such processes, and are associated with poorer future interpersonal, educational, occupational, mental, and physical health outcomes (Clayborne et al. 2019; Copeland et al. 2014; Swan and Kendall 2016). As development is influenced by the environments individuals develop within (Bronfenbrenner 1994), clarifying the role of modifiable, environmental risks is important for intervention development. ACEs and childhood economic disadvantage are two such risk factors for depression and anxiety. However, it is unclear how ACEs and economic disadvantage operate together to influence adolescent outcomes.

### 2.2 Adverse Childhood Experiences (ACEs)

ACEs, including abuse, neglect, and significant parent challenges (e.g., parental mental illness, substance use, incarceration, or intimate partner violence [IPV]), are prevalent and interrelated. Further, cumulative ACEs are a well‐documented risk factor for depression and anxiety (Centers for Disease Control and Prevention 2021; Dong et al. 2004; Elmore and Crouch 2020; Felitti et al. 1998; Kim et al. 2021b; Moore and Stoddard 2024). Much research on cumulative ACEs measures a count of ACEs experienced before age 18; however, this approach is limited (Felitti et al. 1998; Fitzgerald and Gallus 2024; Karatekin et al. 2022). It assumes all ACEs operate through similar pathways with similar magnitude to influence outcomes and suggests developmental timing is insignificant. These assumptions are likely inaccurate in sum.

Recent research indicates ACEs comprise two subdomains: maltreatment (i.e., abuse, neglect) and parent challenges (i.e., parental incarceration, mental/behavioral health challenges) (Moore et al. 2025c). Such subdomains may influence adolescent mental health differently. Several studies have found cumulative maltreatment is a stronger predictor of mental/behavioral health challenges than cumulative parent challenges; however, findings are inconsistent (Fitzgerald and Gallus 2024; Garnsey et al. 2023; Negriff 2020; Sayyah et al. 2022; Wang et al. 2021). One potential explanation for these mixed findings may be that studies examining ACE subdomains largely overlook the developmental timing of ACEs.

Literature suggests cumulative ACEs experienced during early childhood (i.e., ages 0–5) may be more detrimental than cumulative ACEs experienced during other developmental stages (Center on the Developing Child 2007). ACEs can be associated with atypical, harmful stress responses within the body that are linked to maladaptive structural and functional changes to the brain and subsequent mental health challenges (Hill and Belger 2022; McEwen et al. 2016). Despite concerns of elevated risk in early childhood, less research has focused on the effects of cumulative ACEs within this period.

Moreover, the relative impact of distinct ACE subdomains (i.e., maltreatment vs. parent challenges) may vary based on the developmental timing of exposure. Children are completely dependent upon caregivers to meet basic needs in early childhood, and child‐caregiver attachment formation is critical during this period (Ainsworth et al. 2015; Bowlby 1982; Gee 2016) Thus, parent challenges ACEs (that may significantly impede engaged caregiving) may be more harmful during early childhood than other developmental periods when children are more independent and develop/strengthen non‐familial relationships (Doom et al. 2022). Prior studies examining the distinct effects of ACE subdomains without distinguishing early childhood exposures from later exposures may have obscured these differences.

### 2.3 Childhood Economic Status

Low household income is associated with adolescent mental health challenges and ACEs (Reiss 2013; Walsh et al. 2019). Low household income can affect adolescent health and wellbeing through increased familial stress, impaired relationships (e.g., marital conflict, IPV), and harsh parenting practices (e.g., physical punishment, abuse) (Duncan et al. 2017; Yoshikawa et al. 2012). Low income may also influence health and wellbeing through exposure to violent environments and decreased access to health‐promoting resources/services (e.g., access to mental health care), among other factors (Duncan et al. 2017; Yoshikawa et al. 2012). Mechanisms situated within higher‐order ecological levels (e.g., access to resources/services) shape the contexts in which other environments more proximal to the child (e.g., family units) operate. This suggests that economic status may also shape, or moderate, the downstream effects of ACEs. For example, if two children experience the same ACEs, the economically disadvantaged child is hypothesized to experience worse mental health sequelae than their peer because they are less likely to have access to high‐quality mental health care. One existing study reported an interaction between household income and ACEs in relation to adolescent trauma‐related mental health symptoms, finding the association between ACEs and mental health symptoms was stronger for lower‐income youth (Andrews et al. 2015). However, the role of economic disadvantage in the relationship between ACEs and adolescent mental health is understudied (Moore et al. 2025b).

### 2.4 Current Study

To address gaps in existing literature, we (1) examined associations between early childhood ACEs (subindexed as Maltreatment vs. Parent challenges), household income, and adolescent depression and anxiety symptoms, and (2) investigated whether household income moderates the associations between ACEs and depression/anxiety symptoms.

## 3 Materials & Methods

### 3.1 Sample

Data come from the Future of Families and Child Wellbeing Study (FFCWS 2024), a birth cohort study of children born between 1998 and 2000 in 20 large U.S. cities (populations > 200,000). The study oversampled unmarried mothers (three unmarried‐to‐one married), enrolling many participants with minoritized ethnoracial identities and low incomes (see Reichman et al. 2001 for additional information). Parents (primarily biological mothers and fathers) were surveyed at the child's birth (baseline) and when the children were approximately 1, 3, 5, 9, and 15 years old. If a survey was completed at ages 3, 5, or 9, the primary caregiver was recruited for a supplemental in‐home survey for the corresponding year (when some ACEs were measured). In‐home survey response rates were 79%, 81%, and 77%, respectively. Youth were also surveyed at ages 9 and 15 (FFCWS, 2024). At age 15, 73% of the baseline sample remained.

The sample for the current study (*n* = 2523; 52% of the baseline sample) included youth who participated in the age 15 survey and had outcome data available, whose biological mother or father (hereafter, “parents”) was their primary caregiver at ages 1, 3, and 5, and had parent participation in at least the age 3 or age 5 in‐home survey. Six youth were omitted for missing data on anxiety and depression after accounting for other eligibility criteria. Compared to youth excluded from our analyses, our analytic sample had mothers who were significantly younger; more likely to identify as Black, be U.S. born, and graduate from high school; and less likely to identify as Hispanic or other race or be married or cohabiting with the child's father at the child's birth. Our analytic sample also experienced more maltreatment (mean difference [MD] −0.24, *p* < 0.001) and parent challenges (MD −0.26, *p* < 0.001) than excluded participants, but household income (relative to needs) was not significantly different between the two samples. To investigate the impact of our sample restrictions, we conducted a supplemental analysis using the entire baseline sample and full information maximum likelihood estimation techniques to address sample missingness (Supplemental Table 6). Our main findings did not substantially change in this supplemental analysis; rather, the supplemental analysis suggested the estimates in our main analysis were conservative. This study was deemed exempt by the University of Michigan Institutional Review Board.

### 3.2 Measures

**Household Income:** Household income relative to needs (determined by the U.S. Census Bureau's Official Poverty Measure, which accounts for household size and composition) was calculated using parent‐reported household income at baseline. Existing literature indicates a possible pathway linking socioeconomic status at birth to adolescent mental health via early childhood ACEs (Straatmann et al. 2020). Thus, we used baseline income to ensure temporality with our ACEs, in case our findings supported such mediation. Income relative to a household's basic needs, rather than absolute income, provides a clearer picture of a household's financial situation, as it accounts for household size, which affects need. Income was coded as a percentage of the federal poverty level (FPL); 100% FPL indicated income equal to basic needs. Income was categorized as: < 50% FPL (the poorest and reference category), 50‐99% FPL, 100‐199% FPL, 200‐299% FPL, and ≥ 300% FPL. To contextualize the most‐affluent category, incomes ranged from 300% to 1400% of the FPL (median: 480% FPL). The FFCWS imputed incomes for about 25% of participants; we controlled for whether these data were imputed in all of our analyses.

**Adverse Childhood Experiences (ACEs):** Seven ACEs were parent‐reported at ages 1, 3, and 5 and sub‐indexed as Maltreatment (harsh physical discipline, emotional/verbal abuse, neglect) versus Parent Challenges (parental mental illness, problematic substance use, incarceration, IPV). Individual ACEs were dichotomized (1=exposure, 0=no exposure), then summed within their sub‐indices (i.e., Maltreatment, range: 0–3; Parent Challenges, range: 0–4). This delineation of ACEs is supported by previous factor analyses (Afifi et al. 2020; Ford et al. 2014; Mersky et al. 2017; Moore et al. 2025c).

Parent Challenges ACEs were measured at ages 1, 3, and 5 with questions assessing whether either parent met criteria for probable depression or anxiety; struggled with alcohol or drug use that interfered with daily life or met criteria for probable dependence; had been incarcerated; or experienced physical, verbal, or sexual violence. Maltreatment ACEs were measured at ages 3 and 5 with the Parent‐Child Conflict Tactics Scale (CTS) Physical Assault, Psychological Aggression, and Neglect subscales (5‐items each; Straus et al. 1998). For the CTS, item responses captured the frequency of particular events (e.g., the parent shook the child once, twice, 3–5 times, etc.). Following prior literature (Gajos et al. 2023; Moore et al. 2025a), we coded individuals as exposed to each maltreatment ACE if they experienced an item within that subscale six or more times in a year. Supplemental Table 1 provides additional information on ACE variables.

### 3.3 Adolescent Mental Health

***Depression Symptoms.*** Depression symptoms were youth‐reported at age 15 using a 5‐item version of the Center for Epidemiologic Studies Depression Scale (CES‐D; Perreira et al. 2005; Radloff 1977). Items asked youth their level of agreement if, in the past 4 weeks, they felt (1) like they couldn't shake off the blues, even with help; (2) sad; (3) happy; (4) depressed; or (5) like life was not worth living. Response options ranged from (0) strongly agree to (3) strongly disagree. Items were reverse‐coded (except the happiness item) so higher scores indicated worse symptoms, then averaged together (range: 0‐3). The CES‐D has been validated in a racially/ethnically diverse adolescent sample (Perreira et al. 2005) and had adequate reliability here (Cronbach's alpha=0.75).

***Anxiety Symptoms.*** Anxiety symptoms were youth‐reported at age 15 using a 6‐item version of the Brief Symptom Inventory‐18 (BSI‐18; Kim et al. 2021a). Items asked youth their level of agreement if, in the past 4 weeks, they (1) experienced spells of terror/panic; (2) felt tense or keyed up; (3) got suddenly scared for no reason; (4) felt nervous or shaky inside; (5) felt fearful; and (6) felt so restless they couldn't sit still. Response options ranged from (0) strongly agree to (3) strongly disagree. Items were reverse‐coded so higher scores indicated worse symptoms, then averaged together (range: 0–3). The BSI‐18 has been validated across racially/ethnically diverse adolescents (Kim et al. 2021a) and had adequate reliability here (Cronbach's alpha=0.76).

**Demographic Covariates**. Youth covariates included adolescents' age in months at the age 15 survey, youth‐reported race/ethnicity at age 15 (non‐Hispanic Black, non‐Hispanic White, Hispanic, non‐Hispanic multiracial/other), and mother‐reported youth sex at birth (female, male). Additional covariates to account for the oversampling of unmarried mothers included the mother's baseline age, educational attainment, nativity (U.S. born or not), and relationship with the child's biological father (married, cohabiting, neither).

### 3.4 Analyses

Analyses were conducted using Stata version 18.0 (StataCorp 2021). We first calculated correlations of all variables and descriptive statistics. Next, we conducted a series of linear regressions with depression symptoms as the outcome, followed by a separate series of regressions with anxiety symptoms as the outcome. Critical alpha was set at ≤ 0.05.

First in the series of regressions, we tested the mental health outcome against household income and the ACE subindices separately. We then included the ACE sub‐indices and household income together in a single model. Lastly, we added interaction terms for household income and Maltreatment, and household income and Parent Challenges, to assess whether household income moderated the association between ACEs and depression/anxiety symptoms. We also estimated predicted probabilities and marginal effects for each interaction model to better visualize the predicted effects of increasing ACEs on our outcomes for each income strata using all information from the specified model. All covariates, except maternal education (due to a high correlation with income), were included in each model.

Lastly, because we used conservative thresholds to dichotomize Maltreatment ACEs (i.e., indicating exposure to an ACE if an item was experienced ≥ 6 times in a year), we conducted sensitivity analyses where we lowered the threshold for exposure to each Maltreatment ACE to ≥ 3 experiences in a year. We could not lower the threshold further because a substantial proportion of youth experienced many Maltreatment ACE indicators at low frequencies, resulting in limited variation to study.

## 4 Results

Adolescents (*n* = 2523) were approximately 51% female and 51% non‐Hispanic Black. The Maltreatment and Parent Challenges sub‐indices were not highly correlated (r = 0.15). Maternal baseline education was highly correlated with household income (r = 0.50); therefore, we omitted maternal education from analyses. Approximately 7% of adolescents experienced no early childhood ACEs, 16% experienced one, 31% experienced two, 25% experienced three, and 21% experienced four or more. Maltreatment ACEs were more prevalent than Parent Challenges ACEs, and anxiety symptoms were more prevalent than depression symptoms. Approximately 36% of adolescents were born to households experiencing poverty. See Table 1 for additional information.

**Table 1:** Descriptive statistics of the analytic sample (*n* = 2523).

Characteristics	Value
Depressive Symptoms, median (IQR)	0.40 (0.0, 1.0)
Anxiety Symptoms, median (IQR)	0.67 (0.33, 1.33)
Individual ACEs, no. (%)	
Harsh Physical Discipline	1489 (59.0)
Emotional/Verbal Abuse	2113 (83.8)
Neglect	86 (3.4)
Parent Substance Use	228 (9.0)
Parent Mental Illness	976 (38.7)
Parent Incarceration	602 (23.9)
Parent Intimate Partner Violence	710 (28.1)
ACE Indices, mean (SD)	
Maltreatment Score	1.5 (0.8)
Parent Challenges Score	1.0 (1.0)
Total ACE Score	2.5 (1.3)
Household Income, no. (%)	
≥ 300% FPL	561 (22.2)
200–299% FPL	381 (15.1)
100–199% FPL	682 (27.0)
50–99% FPL	437 (17.3)
< 50% FPL	462 (18.3)
Youth Race/Ethnicity, no. (%)	
White, non‐Hispanic	419 (17.5)
Black, non‐Hispanic	1233 (51.4)
Hispanic/Latino	568 (23.7)
Multiracial/Other, non‐Hispanic	181 (7.5)
Youth Sex, no. (%)	
Male	1228 (48.7)
Female	1295 (51.3)
Youth Age in months, mean (SD)	186.7 (7.9)
Mother Baseline Age in years, mean (SD)	25.0 (5.9)
Mother Baseline Education, no. (%)	
College or graduate school	252 (10.0)
Some college or technical school	653 (25.9)
High school or equivalent	786 (31.2)
Less than high school	828 (32.9)
Mother Immigration Status, no. (%)	
U.S. born	2202 (87.6)
Foreign born	313 (12.5)
Parent Baseline Relationship Status, no. (%)	
Married	567 (22.5)
Cohabitating	912 (36.2)
Not Married or Cohabiting	1044 (41.4)

*Note:* Maternal characteristics were assessed at the baseline survey (at the child's birth). For the individual ACEs, only numbers for youth experiencing those ACEs are shown; the alternative option for each ACE was no exposure.

Abbreviations: %, percent of sample; ACEs, Adverse Childhood Experiences; IQR, interquartile range; no., number of respondents; SD, standard deviation.

### 4.1 Depression Symptoms

Results for depression and anxiety symptoms are detailed in Table 2 (with complete results, including those for demographic covariates, detailed in Supplementary Tables 2 and 3). In the model including only household income (model 1), higher income (≥ 300% FPL) was associated with fewer depression symptoms compared to incomes < 50% FPL (standardized beta, β = −0.11, standard error [SE] = 0.04). In the model including only Maltreatment (model 2), Maltreatment was not associated with depression symptoms. In the model including only Parent Challenges (model 3), Parent Challenges *were* associated with more depression symptoms (β = 0.06; SE = 0.01). When Maltreatment and Parent Challenges were included in the same model (model 4), Parent Challenges remained the only significant predictor (β = 0.06, SE = 0.01). In the model including income, Maltreatment, and Parent Challenges (model 5), household incomes ≥ 300% FPL (compared to incomes < 50% FPL) were associated with fewer depression symptoms (β = −0.09, SE = 0.04), and Parent Challenges were associated with more depression symptoms (β = 0.6, SE = 0.01). In the interaction model (model 6), household income did not moderate the effects of either ACE sub‐index. However, the stratified predicted probabilities and marginal effects (Table 3) indicated the Parent Challenges sub‐index was only associated with depression symptoms for youth with household incomes < 100% FPL (the change in y due to a marginal change in x [dy/dx] = 0.08, *p* < 0.05). Differences in significance based on household income were not found for the Maltreatment sub‐index.

**Table 2:** Independent and Interactive Effects (with standard errors) of Adverse Childhood Experiences (ACEs) and Household Income on Adolescent Depression (top) and Anxiety (bottom).

	Model 1	Model 2	Model 3	Model 4	Model 5	Model 6
Depressive Symptoms (*n* = 2351)						
Household Income						
< 50% FPL	(ref)				(ref)	(ref)
50–99% FPL	−0.02 (0.04)				−0.01 (0.04)	−0.06 (0.10)
100–199% FPL	−0.05 (0.04)				−0.05 (0.04)	−0.00 (0.09)
200–299% FPL	−0.06 (0.04)				−0.04 (0.04)	−0.07 (0.10)
≥ 300% FPL	−0.11 (0.04)**				−0.09 (0.04)*	−0.00 (0.09)
ACE Indices						
Maltreatment		0.02 (0.02)		0.01 (0.02)	0.01 (0.02)	0.00 (0.04)
Parent Challenges			0.06 (0.01)***	0.06 (0.01)***	0.06 (0.01)***	0.07 (0.03)*
Interactions						
Maltreatment (CM) × Household Income						
CM × 0–49% FPL						(ref)
CM × 50–99% FPL						0.02 (0.05)
CM × 100–199% FPL						−0.01 (0.05)
CM × 200–299% FPL						0.04 (0.05)
CM × ≥ 300% FPL						0.01 (0.05)
Parent Challenges (PC) × Household Income						
PC × 0–49% FPL						(ref)
PC × 50–99% FPL						0.01 (0.04)
PC × 100–199% FPL						−0.02 (0.04)
PC × 200‐299% FPL						−0.03 (0.04)
PC × ≥ 300% FPL						−0.01 (0.04)
Anxiety Symptoms (*n* = 2389)						
Household Income						
< 50% FPL	(ref)				(ref)	(ref)
50–99% FPL	−0.02 (0.05)				−0.01 (0.05)	0.00 (0.10)
100–199% FPL	−0.07 (0.04)^†^				−0.06 (0.04)	0.04 (0.09)
200–299% FPL	−0.08 (0.05)				−0.06 (0.05)	0.03 (0.11)
≥ 300% FPL	−0.09 (0.05)^†^				−0.06 (0.05)	−0.03 (0.10)
ACE Indices						
Maltreatment		0.02 (0.02)		0.01 (0.02)	0.01 (0.02)	−0.01 (0.04)
Parent challenges			0.06 (0.01)***	0.06 (0.01)***	0.06 (0.01)***	0.12 (0.03)***
Interactions						
Maltreatment (CM) × Household Income						
CM × 0–49% FPL						(ref)
CM × 50–99% FPL						0.06 (0.06)
CM × 100–199% FPL						0.00 (0.05)
CM × 200–299% FPL						0.03 (0.06)
CM × ≥ 300% FPL						0.02 (0.06)
Parent Challenges (PC) × Household Income						
PC × 0–49% FPL						(ref)
PC × 50–99% FPL						−0.08 (0.04)^†^
PC × 100–199% FPL						−0.09 (0.04)*
PC × 200–299% FPL						−0.12 (0.05)*
PC × ≥ 300% FPL						−0.05 (0.04)

*Note:* Bolded values with indicate statistical significance at *p* < 0.05; ***p < 0.001; **p < 0.01; *p < 0.05; ^†^ *p* < 0.10.

Demographic covariates were included in all models but are not shown here; full tables can be found in the Supplemental Materials (Supplemental Tables 2 & 3).

There was a significant difference in the effect sizes of household income ≥ 300% FPL versus Parent Challenges in relation to depression symptoms in postestimation comparisons

Abbreviations: ACEs, Adverse Childhood Experiences; CM, child maltreatment; FPL, federal poverty level; PC, parent challenges.

**Table 3:** Marginal effects (or dy/dx, indicating the change in outcome associated with a minute change in exposure) and standard errors for increasing Maltreatment & Parent Challenges by Income Category.

Income	Depression		Anxiety	
	CM	PC	CM	PC
< 50% FPL	0.00 (0.04)	0.07 (0.03)*	−0.01 (0.04)	0.12 (0.03)***
50–99% FPL	0.02 (0.04)	0.08 (0.03)*	0.05 (0.04)	0.04 (0.03)
100–199% FPL	−0.01 (0.03)	0.05 (0.03)^†^	−0.01 (0.03)	0.03 (0.03)
200–299% FPL	0.04 (0.04)	0.04 (0.03)	0.02 (0.04)	0.01 (0.04)
≥ 300% FPL	0.01 (0.04)	0.05 (0.03)^†^	0.02 (0.04)	0.07 (0.03)*

*Note:* ***p < 0.001; *p < 0.5; †*p* < 0.10.

Abbreviations: CM, child maltreatment sub‐index; FPL, federal poverty level; PC, parent challenges sub‐index.

### 4.2 Anxiety Symptoms

In the model including only household income (model 1), income was not associated with anxiety symptoms. In the model including only Maltreatment (model 2), Maltreatment was not associated with anxiety symptoms. In the model including only Parent Challenges (model 3), Parent Challenges *were* associated with more anxiety symptoms (β = 0.06; SE = 0.01). When Maltreatment and Parent Challenges were included in the same model (model 4), Parent Challenges remained the only significant predictor of anxiety symptoms (β = 0.06, SE = 0.01). In the model including income, Maltreatment, and Parent Challenges (model 5), only Parent Challenges were significantly associated with anxiety symptoms (β = 0.6, SE = 0.01). In the interaction model (model 6), youth with incomes at 100–199% FPL and 200–299% FPL experienced fewer anxiety symptoms than youth with incomes < 50% FPL within the context of increasing Parent Challenges. No significant difference was identified between youth with incomes < 50% FPL and ≥ 300% FPL or between < 50% FPL and 50‐99% FPL. The predicted probabilities and marginal effects (Table 3) indicated the Parent Challenges sub‐index was only associated with more anxiety symptoms for youth with household incomes < 50% FPL and ≥ 300% FPL (dy/dx: 0.12, *p* < 0.001 and dy/dx = 0.07, *p* < 0.05; respectively). Differences in significance based on household income were not found for Maltreatment.

### 4.3 Sensitivity Analysis

After lowering the threshold used to dichotomize Maltreatment ACEs, results for anxiety symptoms were similar to those of the original analyses, but there were differences for depression symptoms (see Supplemental Tables 4 and 5). Cumulative Maltreatment became a significant predictor of depression symptoms when analyzed in a model by itself (model 2), but was no longer significant when analyzed simultaneously with Parent Challenges (model 3). The interaction model (model 6) revealed a new, significant interaction between household income and Maltreatment, where youth with household incomes < 50% FPL experienced more depression symptoms within the context of greater Maltreatment than their peers with household incomes at 100–199% FPL. Stratified marginal effects of the interaction model suggested that Maltreatment was only associated with depression symptoms for youth in the lowest income category (< 50% FPL), with an overall U‐shaped data pattern across all income categories, similar to those found in the original analyses.

## 5 Discussion

The current study examined associations between *early childhood* ACEs (sub‐indexed as Maltreatment vs. Parent Challenges) and household income in relation to adolescent depression and anxiety symptoms and investigated whether household income moderated the associations between ACEs and adolescent outcomes. We found the Parent Challenges sub‐index, but not the Maltreatment sub‐index, predicted both anxiety and depression symptoms. Household income was only associated with depression symptoms, yet emerged as a stronger predictor than the Parent Challenges sub‐index. Interaction effects (and predicted probabilities) indicated that increasing Parent Challenges was only significantly associated with depression and anxiety symptoms for individuals within specific income strata.

### 5.1 ACEs and Mental Health

Our findings bolster well‐documented associations between ACEs and adolescent mental health challenges (Petruccelli et al. 2019), but our Parent Challenges sub‐index emerged as a stronger predictor than our Maltreatment sub‐index, which contrasts with previous literature (Garnsey et al. 2023; Negriff 2020; Sayyah et al. 2022). However, few prior studies investigate the distinct effects of cumulative maltreatment versus parent challenges, and they do not distinguish ACEs experienced during *early childhood* from ACEs experienced at other developmental stages, as we did. Overall, these findings suggest that previous evidence linking a single composite ACE index with poor mental health could be largely driven by a subset of ACEs. Continued research on how distinct subdomains of adversity experienced within distinct developmental windows may uniquely influence different outcomes is warranted.

One possible explanation for the prominent role of Parent Challenges in this study is that the sub‐index may better capture youths' cumulative exposure to parental separation/disengagement, which is particularly detrimental in early childhood. Very young children biologically and psychologically require stable, responsive, and sensitive relationships, facilitated by regular proximity to and communication with their caregiver(s) for healthy development, especially the development of self‐regulation and coping skills (Ainsworth et al. 2015; Bowlby 1982; Fitton 2012). Our Parent Challenges sub‐index comprises experiences of literal separation (i.e., parent incarceration) and parent challenges that likely inhibit caregiver engagement with their child (i.e., mental illness, problematic substance use). Moreover, experiences of parental incarceration or mental/behavioral health challenges may be more chronic than maltreatment experiences (Bigler et al. 2025). Multiple parent challenges may represent increased or longer‐lasting instability and decreased engagement in the child‐caregiver relationship, leading to altered child development and increased risk of adolescent mental health challenges beyond the risk associated with cumulative maltreatment experiences *in early childhood*. Existing literature on the effects of parenting behaviors on child mental health somewhat supports this notion. Doom et al. (2022) reported parental disengagement at age 1 predicted an atypical childhood stress response and adolescent depression symptoms; whereas parental harshness in adolescence predicted an atypical adolescent stress response and anxiety symptoms. Relatedly, Bayer et al. (2006) found that parenting characterized as low warmth/engagement (e.g., behaviors like limited parental involvement or limited expressions of affection toward the child) was associated with early childhood internalizing symptoms, but there was no association with power‐assertive/punitive parenting (e.g., behaviors like yelling at, spanking, slapping, or hitting the child).

Our study design also differed from previous studies in other ways that may have contributed to our incongruent findings. Previous studies that reported maltreatment ACEs were a stronger predictor of adolescent mental health (vs. parent/household challenges) included sexual abuse as an ACE (Garnsey et al. 2023; Negriff 2020; Sayyah et al, 2021). Such information was not available for the current study. It is possible that if we had included this type of maltreatment, the risk of adolescent mental health challenges associated with the Maltreatment sub‐index may have substantially increased and surpassed the risk of cumulative Parent Challenges. However, previous research does not strongly support this notion (Dye 2019; Mills et al. 2013; Nelson et al. 2017; Radell et al. 2021). Yet, this possibility should be considered in future research. There was also a difference in the level of measurement detail for Maltreatment variables. In previous studies, respondents self‐reported abuse or neglect more generally, without identifying perpetrators. Whereas our study captured maltreatment perpetrated by parents. Similarly, prior studies included challenges within the household broadly (e.g., has *anyone* in the household struggled with mental illness vs. *parents* specifically), which may have unique implications for development in early childhood (Garnsey et al. 2023; Negriff 2020; Sayyah et al. 2022). For example, attachment theory posits that the incarceration of a child's caregiver would be more harmful to a child than the incarceration of a co‐resident adult. This could explain, at least partially, the prominent effects observed for our Parent Challenges sub‐index.

It is also important to consider the measurement of our Maltreatment ACEs when interpreting our null findings. Our sensitivity analysis revealed a difference between our primary and sensitivity analyses regarding the association between the Maltreatment sub‐index and adolescent depression when Maltreatment was assessed *individually*. Yet, in both analyses, when the Maltreatment and Parent Challenges sub‐indices were examined *simultaneously*, Maltreatment was a non‐significant predictor. However, we cannot completely rule out cumulative early childhood maltreatment as playing a role in adolescent depression or anxiety for several reasons. Most of our sample experienced some/low levels of maltreatment. Therefore, our ability to test the effects of a Maltreatment sub‐index based on *any* exposure (vs. frequent/chronic exposure) to the individual ACEs was limited. Despite such saturation, we also cannot rule out the possibility that parents underreported maltreatment. Further, the conventional approach of dichotomizing ACEs limits our ability to thoroughly capture the diverse range of possible maltreatment experiences, which may have influenced results (McGuire et al. 2024). Additional research is needed to confirm our null findings.

Another important consideration is our sample's composition. Participants reported more ACEs than other samples and the U.S. population and included a larger proportion of youth identifying as Black or Hispanic, born to low‐income households and born to unmarried mothers (Garnsey et al. 2023; Negriff 2020; Sayyah et al, 2021; United States Census Bureau 2024). These demographic differences may have influenced our findings. Additional research with alternative samples (including similarly diverse and nationally representative samples) is needed to validate and clarify the generalizability of our findings.

### 5.2 The Role of Household Income

Our findings partially align with existing literature linking lower household income with adolescent mental health challenges. In the current study, lower household incomes were only associated with depression symptoms (not anxiety). Youth with household incomes below the FPL (vs. incomes ≥ 300% FPL) reported greater depression symptoms. However, the effect of household income was attenuated in magnitude after accounting for ACEs, which suggests ACEs may be a mediator between low household income and adolescent depression. This aligns with previous, yet limited, literature reporting cumulative ACEs are a partial mediator through which low household income/resources influence adolescent health and wellbeing (Barnhart et al. 2022; Straatmann et al. 2020). Of note, prior mediation studies did not consider maltreatment and parent challenges ACEs separately, as we did. While formal mediation analysis is outside the scope of the current study, we recommend continued research into this relationship to better understand these pathways. Despite the attenuation in magnitude, we highlight that very low household incomes (< 50% FPL) remained the strongest predictor for adolescent depression symptoms compared to both ACE sub‐indices. While addressing early childhood ACEs may reduce the burden of adolescent depression, our results suggest a need to address the financial hardship of families caring for very young children when considering comprehensive adolescent depression prevention strategies.

Although household income was not associated with adolescent anxiety symptoms, income moderated the association between Parent Challenges and anxiety symptoms. Youth with the lowest and highest household incomes compared to youth with middle‐range incomes reported more anxiety symptoms within the context of increasing Parent Challenges. There was no difference between youth with the lowest and highest incomes. These findings (along with the predicted probabilities and marginal effects) suggest a U‐shaped pattern between household income and anxiety symptoms within the context of increasing Parent Challenges. There was no significant interaction found between household income and Maltreatment for either outcome, and income did not moderate the association between Parent Challenges and adolescent depression symptoms. However, the predicted probabilities and marginal effects for this latter interaction suggested a similar U‐shaped patterning to that found for anxiety symptoms. While unexpected, this U‐shaped patterning for mental health challenges has been identified in emerging research on adult income and mental illness (Li et al. 2022). Further, it helps to explain the somewhat contradictory evidence that indicates youth in low‐income *and* affluent households exhibit greater mental health challenges than their more or less financially‐advantaged peers, respectively (Luthar 2003; Reiss 2013). Existing literature suggests there may be differing mechanisms by which youth at each end of the economic spectrum are at increased risk of mental health challenges, as described below.

The finding that youth from very low‐income households experienced the negative effects of accumulating Parent Challenges more strongly than more‐advantaged youth aligns with our understanding that economic hardship influences mental health, in part, through institutional factors, like decreased access to mental health care. Given the many overlapping layers of systemic disadvantage, youth from lower‐income households were hypothesized to have worse outcomes than youth from higher‐income households. While we did not observe a similar moderating effect for cumulative maltreatment, we attribute this to the overall null association between the Maltreatment sub‐index and adolescent depression/anxiety symptoms in our sample. Alternatively, we consider that each Parent Challenges ACE represents substantial instability for a family, specifically the child‐caregiver relationship, as previously discussed. Families experiencing poverty are navigating an additional and unique type of instability that may interact synergistically with the instability of cumulative Parent Challenges, thereby resulting in worse outcomes for doubly‐exposed youth.

The finding that youth from the most affluent households in our sample experienced the effects of accumulating Parent Challenges more strongly than youth with more moderate incomes aligns with the existing, albeit limited, literature on affluence and youth mental health challenges (Luthar et al. 2013). While sparse, previous literature describes a “culture of affluence” that values autonomy, personal control, perfectionism, and privacy (Haselschwerdt and Hardesty 2017; Luthar 2003; Luthar et al. 2013). Further, affluent communities are often characterized as hyper‐achieving, extremely competitive and materialistic, with the pursuit of wealth and status coming at the cost of personal relationships (Luthar et al. 2013). Supportive extra‐familial relationships may be limited for parents and adolescents as competition is prioritized over interdependence (e.g., avoiding asking for help for fear of decreased social standing) and competitive, resume‐building activities replace leisurely community gatherings (e.g., competitive sports leagues vs. impromptu games at the park) (Luthar et al. 2013). Intrafamilial relationships, including caregiver‐child attachments, may be weakened as parents may be highly involved in their children's lives to ensure “success” while placing less emphasis on nurturing relationships. Additionally, parents may be less emotionally available and larger houses enable physical isolation (Luthar 2003; Luthar et al. 2013). Disrupted caregiver‐child attachments may compound upon the similar attachment‐disrupting effects of Parent Challenges ACEs, thereby producing synergistic effects. We also posit that stigma may exacerbate the mental health effects of ACEs. A stronger internal locus of control paired with competitive, perfectionistic communities increases the possibility that significant parental challenges will be hidden/unaddressed (e.g., parents unwilling to seek behavioral health care due to reputational concerns), but that affected individuals and families will perceive a greater sense of personal failure from these challenges (Luthar et al. 2013). Such stigma in high‐income populations has been documented regarding mental/behavioral health challenges (Foster 2021) and IPV (Haselschwerdt and Hardesty 2017). Overall, findings indicate a need to formally interrogate the mechanisms by which household income moderates the effects of early childhood ACEs on adolescent depression/anxiety outcomes.

### 5.3 Strengths and Limitations

While we used the conventional approach of operationalizing ACEs as count indices, such measures lack information on severity and frequency, which could provide a more comprehensive understanding of ACE effects. We did not have information on sexual abuse, and it is possible other Maltreatment ACEs were underreported because parents self‐reported their behaviors. These limitations may explain why we did not observe an association between early childhood maltreatment and adolescent depression/anxiety, despite substantial literature documenting the mental health effects of maltreatment (Baldwin et al. 2023). Thus, the null effect observed should be interpreted cautiously. Further, future studies should consider incorporating objective data (e.g., administrative data from Child Protective Services or medical records) to complement self‐reported data for both ACEs and mental health symptoms, which may be underreported due to social desirability bias. Additionally, the generalizability of our findings is limited given our sample was not nationally representative and included a single cohort of youth. Moreover, a major economic downturn (the Great Recession) occurred when participants were approximately 9 years old, which may have influenced adolescent mental health. Additional research is needed on the associations examined in this study within other cohorts of youth. Yet, data were collected prospectively, which is a strength for ACEs research. The current study is also strengthened by its use of longitudinal data collected from a large sample, comprised of a substantial proportion of participants with demographics traditionally underrepresented in research.

### 5.4 Implications for Policy and Practice

Our findings (in combination with existing literature) support that primary ACE‐prevention interventions are urgently needed as part of a comprehensive strategy to promote healthy child development and adolescent mental health. Moreover, our findings highlight a specific need for more effective strategies to identify and support parents of very young children (or expectant parents) who are struggling with mental/behavioral health challenges, IPV, criminalized behavior, and/or poverty, to support whole‐family wellbeing and limit children's exposure to harmful adversity. The Centers for Disease Control and Prevention (2019) has promoted a multi‐system approach to addressing ACEs, including policies that strengthen families' economic supports, address social norms around violence, promote early childhood services and skills training for parents, and increase health care access for all. Health professionals, particularly nurses, who belong to the most trusted profession in the U.S. (American Nurses Association 2024), are ideal advocates for such policies. Although nurses can also play a more direct role in ACE prevention/intervention efforts by promoting trauma‐informed environments across practice settings, acknowledging that childhood adversity is prevalent and can have long‐lasting consequences, identifying parents struggling with significant challenges (e.g., mental illness) and encouraging them to engage with supportive services.

## 6 Conclusion

In conclusion, we found early childhood Maltreatment and Parent Challenges ACEs were differentially associated with adolescent depression and anxiety symptoms, with some associations moderated by household income. Future research should further investigate these associations to better understand how specific dimensions of childhood adversity, when experienced in specific developmental periods, may be driving well‐documented associations between cumulative ACEs and health/wellbeing outcomes. Policies that support caregivers of very young children, specifically addressing parental incarceration, mental health/substance use problems, IPV, and/or poverty, may help promote child and adolescent health and well‐being. Effective ACE prevention strategies are urgently needed. Health professionals are well‐positioned to advocate for supportive policies, promote trauma‐informed environments, and identify/address significant parental challenges early in children's development.

## Author Contributions

**Chelsea R. Moore:** conceptualization, funding acquisition, methodology, data curation, formal analysis, visualization, writing – original draft, writing‐ review and editing. **Philip T. Veliz:** methodology, formal analysis, supervision, writing – review and editing. **Alison L. Miller:** conceptualization, supervision, writing – review and editing. **Natasha V. Pilkauskas:** conceptualization, data curation, supervision, writing – review and editing. **Todd I. Herrenkohl:** conceptualization, supervision, writing – review and editing. **Sarah A. Stoddard:** conceptualization, funding acquisition, methodology, data curation, formal analysis, visualization, writing – review and editing. All authors read and approved the final manuscript.

## Ethics Statement

This study was deemed exempt by the University of Michigan Institutional Review Board.

## Conflicts of Interest

The authors have no relevant financial or non‐financial interests to disclose.

## Supporting information


Supporting File

## Data Availability

We use publicly available data that can be accessed upon application from the Future of Families and Child Wellbeing Study (FFCWS) website (https://ffcws.princeton.edu/).
