# Supplementary material for: Individual and Interactive Effects of Adverse Childhood Experiences and Household Income on Adolescent Depression and Anxiety Symptoms
Source: Res Nurs Health. 2026 Jul 18;49(5):744–55. doi: 10.1002/nur.70107 (PMC13539674; doi:10.1002/nur.70107)
Supplement: Supplementary file 1 [file NUR-49-744-s001.pdf]

Supplemental Materials

Supplemental Table 1. ACE variable construction details.

ACE	Age	Reporter	Question	Response Options	Special Notes
**Harsh Physical Punishment**<br><br>(Conflict Tactics Scale [CTS]- Physical Assault subscale)	3	Primary Caregiver	Past year, times PCG/SCG shook child	(0) This has never happened<br>(1) Once<br>(2) Twice<br>(3) 3-5 times<br>**(4) 6-10 times**<br>**(5) 11-20 times**<br>**(6) More than 20 times**<br>(7) Yes, but not in the past year	Youth experiencing HPP at age 3 = 1,148 (54.0%)… broken down as:<br>• Shook: 19 (0.9%)<br>• Hit on bottom: 271 (12.8%)<br>• Spanked: 941 (44.3%)<br>• Slapped: 779 (36.6%)<br>• Pinched: 64 (3.0%)
			Past year, times PCG/SCG hit child on the bottom with belt or hard object		
			Past year, times PCG/SCG spanked child on bottom with bare hand		
			Past year, times PCG/SCG slapped child on the hand, arm, or leg		
			Past year, times PCG/SCG pinched child		
	5	Primary Caregiver	Past year, times PCG/SCG shook child		Youth experiencing HPP at age 5 = 906 (45.1%)… broken down as:<br>• Shook: 23 (1.1%)<br>• Hit on bottom: 284 (14.1%)<br>• Spanked: 696 (34.6%)<br>• Slapped: 516 (25.7%)<br>• Pinched: 66 (3.3%)
			Past year, times PCG/SCG hit child on the bottom with belt or hard object		
			Past year, times PCG/SCG spanked child on bottom with bare hand		
			Past year, times PCG/SCG slapped child on the hand, arm, or leg		
			Past year, times PCG/SCG pinched child		
**Neglect**<br><br>(CTS- Neglect subscale)	3	Primary Caregiver	Past year, times PCG had to leave child home alone	(0) This has never happened<br>(1) Once<br>(2) Twice<br>(3) 3-5 times<br>**(4) 6-10 times**<br>**(5) 11-20 times**<br>**(6) More than 20 times**<br>(7) Yes, but not in the past year	Youth experiencing neglect at age 3 = 54 (2.5%) … broken down as:<br>• Alone: 6 (0.3%)<br>• Food: 12 (0.6%)<br>• Medical: 4 (0.2%)<br>• Drunk/High: 5 (0.2%)<br>• Loved: 35 (1.7%)
			Past year, times PCG wasn't able to make sure child got food needed		
			Past year, times PCG wasn't able to take child got to a doctor/hospital		
			Past year, times PCG was so drunk/high they had problem taking care of the child		
			Past year, times PCG wasn't able to show or tell child you loved child		
	5	Primary Caregiver	Past year, times PCG had to leave child home alone		Youth experiencing neglect at age 5 = 33 (1.7%) … broken down as:
			Past year, times PCG wasn't able to make sure child got food needed		

	3		Past year, times PCG wasn't able to take child to a doctor/hospital		• Alone: 8 (0.4%)<br>• Food: 7 (0.4%)<br>• Medical: 1 (0.1%)<br>• Drunk/High: 2 (0.1%)<br>• Loved: 16 (0.8%)
			Past year, times PCG was so drunk/high they had problem taking care of child		
			Past year, times PCG wasn't able to show or tell child you loved child		
**Emotional/ Verbal Abuse**<br><br>(CTS-Psychological Aggression subscale)	3	Primary Caregiver	Past year, times PCG/SCG shouted, yelled, or screamed at child	(0) This has never happened<br>(1) Once<br>(2) Twice<br>(3) 3-5 times<br>**(4) 6-10 times**<br>**(5) 11-20 times**<br>**(6) More than 20 times**<br>(7) Yes, but not in the past year	Youth experiencing EVA at age 3 = 1,592 (74.9%) … broken down as:<br>• Shout: 1,193 (56.1%)<br>• Swore: 174 (8.2%)<br>• Send away: 45 (2.1%)<br>• Threat: 1,364 (64.2%)<br>• Dumb/Lazy: 26 (1.2%)
			Past year, times PCG/SCG swore or cursed at child		
			Past year, times PCG/SCG said would send child away or kick out of house		
			Past year, times PCG/SCG threatened to spank/hit child but did not		
			Past year, times PCG/SCG called child dumb or lazy or similar name		
	5	Primary Caregiver	Past year, times PCG/SCG shouted, yelled, or screamed at child		Youth experiencing EVA at age 5 = 1,564 (77.9%) … broken down as:<br>• Shout: 1,245 (61.9%)<br>• Swore: 204 (10.2%)<br>• Send away: 59 (2.9%)<br>• Threat: 1,308 (65.1%)<br>• Dumb/Lazy: 50 (2.5%)
			Past year, times PCG/SCG swore or cursed at child		
			Past year, times PCG/SCG said would send child away or kick out of house		
			Past year, times PCG/SCG threatened to spank/hit child but did not		
			Past year, times PCG/SCG called child dumb or lazy or similar name		
**Parent Problematic Substance Use**	1	Mother & Father	Since child's birth, has drinking/drugs interfered with how you manage daily?	(0) no<br>**(1) yes**	Youth experiencing parent problematic substance use at age 1 = 106 (4.2%)
			Since child's birth, has drinking/drugs interfered with your personal relationships?		
			Since child's birth, have you sought help/been treated for a drug/alcohol problem?		
	3	Mother & Father	Constructed - Mother/father alcohol dependence (CIDI)<br>• Determined by 10-11 items (varies by city)<br>• Example items: largest number of drinks consumed in a day; drinking interferes with life; experienced emotional/psychological problems from alcohol; were under the	(0) no<br>**(1) yes**	Youth experiencing parent problematic substance use at age 3 = 116 (4.6%)

			influence of alcohol in a dangerous situation		
			Constructed - Mother/father drug dependence (CIDI)<br>• Determined by 19 items<br>• Example items: use of drugs (e.g. LSD/ hallucinogens, heroin, cocaine, or sedatives); drug use interfered with work/home/school life; experienced emotional/psychological problems from drug use; were under the influence of drugs in a dangerous situation		
	5	Mother & Father	Past year, did your use of substances interfere with work at school/job/home	(0) no<br>**(1) yes**	Youth experiencing parent problematic substance use at age 5 = 36 (1.4%)
**Parent Mental Illness (i.e., depression or anxiety**)<br><br>(Composite International Diagnostic Interview-Short Form; CIDI-SF)	1	Mother & Father	Constructed - Mother/father meets depression criteria (conservative) (CIDI)<br>• Determined by 11-15 items (varies by city)<br>• Example items: sad/depressed for 2+ weeks in row; lost interest in things that were pleasurable; less energy than usual; weight loss/gain; sleep disturbances	(0) no<br>**(1) yes**	Youth experiencing parent mental illness at age 1 = 482 (19.1%)
			Constructed - Mother/father meets anxious criteria (CIDI)<br>• Determined by 20-24 items (varies by city)<br>• Example items: tense/anxious for 1+ month; worrying a lot more than most other people; easily fatigued; trouble concentrating		
	3	Mother & Father	Constructed - Mother/father meets depression criteria (conservative) (CIDI)<br>• Determined by same items described above	(0) no<br>**(1) yes**	Youth experiencing parent mental illness at age 3 = 556 (22.0%)
			Constructed- Mother/father meets anxiety criteria<br>• Determined by 24 items<br>• Example items: worried/tense/anxious for 1+ month; worrying a lot more than most people; difficult to stop worrying; different worries on your mind at the same time		
	5	Mother & Father	Constructed - Mother/father meets depression criteria (conservative) (CIDI)<br>• Determined by same items described above	(0) no<br>**(1) yes**	Youth experiencing parent mental illness at age 5 = 426 (16.9%)
**Parent Incarceration**	1	Father	Constructed - father is in jail at age 1 interview	(0) no<br>**(1) yes**	Youth experiencing parent incarceration at age 1 = 151 (6.0%)
		Mother	Constructed - father is in jail at age 1 interview		
			Reason for [your] most recent separation from child?	Multiple options…<br>**(4) mom jailed**	

			Is father currently working/in school/ unemployed/in jail?	Multiple options…<br>**(3) in jail**	
	3	Mother & Father	Constructed - father is in jail at either (mother or father) age 3 interview	(0) no<br>**(1) yes**	Youth experiencing parent incarceration at age 3 = 209 (8.3%)
		Father	Is mother currently in jail?		
	5	Mother & Father	Constructed - father is in jail at either (mother or father) age 5 interview	(0) no<br>**(1) yes**	Youth experiencing parent incarceration at age 5 = 520 (20.6%)
		Father	Has mother spent any time in jail in the last 2 years?		
			Is mother currently in jail?		
		Mother	Has father spent any time in jail in the past 2 years?		
**Parent Intimate Partner Violence**	1	Mother	How often does father (or current partner) insult or criticize you?	**(1) Often**<br>(2) Sometimes<br>(3) Never	Youth experiencing parental IPV at age 1 = 340 (13.5%)
			How often does father (or current partner) slap or kick you?		
			How often does father (or current partner) hit you with fist or dangerous object?		
			How often does father (or current partner) force you to have sex/do sexual things?		
			Were you ever cut/bruised/seriously hurt in fight with father (or current partner)?	(0) no<br>**(1) yes**	
		Father	How often does mother insult or criticize you?	(1) Often<br>(2) Sometimes<br>(3) Never	
	3	Mother	How often does father (or current partner) insult or criticize you or your ideas?	**(1) Often**<br>(2) Sometimes<br>(3) Never	Youth experiencing parent IPV at age 3 = 158 (6.3%)
			How often does father (or current partner) slap or kick you?		
			How often does father (or current partner) hit you with fist or an object that can hurt you?		
			How often does father (or current partner) try to make you have sex or do sexual things?		
		Father	How often does mother insult or criticize you or your ideas?		
			How often does mother slap or kick you?		

			How often does mother hit you with fist or an object that can hurt you?		
			How often does mother try to make you have sex or do sexual things?		
	5	Mother	How often does father (or current partner) insult or criticize you or your ideas?	**(1) Often**<br>(2) Sometimes<br>(3) Never	Youth experiencing parent IPV at age 5 = 355 (14.1%)
			How often does father (or current partner) insult/criticize you for not taking good care of the child/home?		
			How often does father (or current partner) slap or kick you?		
			How often does father (or current partner) hit you with a fist or an object that could hurt you?		
			How often does father (or current partner) try to make you have sex or do sexual things you don't want?		
			How often does father (or current partner) throw something at you?		
			How often does father (or current partner) push, grab, or shove you?		
		Father	How often does mother insult or criticize you or your ideas?		
			How often does mother insult/criticize you for not taking good care of the child/home?		
			How often does mother slap or kick you?		
			How often does mother hit you with a fist or an object that could hurt you?		
			How often does mother try to make you have sex or do sexual things you don't want?		
			How often does mother throw something at you?		
			How often does mother push, grab, or shove you?		

Note: For the Response Options column, bolded responses represent the threshold at which an individual item triggered that ACE to be coded as 1 (versus 0, meaning no exposure to that ACE). For example, Harsh Physical Punishment was coded as 1 (versus 0) if a parent reported the child was hit on the bottom with a belt or hard object 6 or more times in the past year at age 3 or age 5. Variable descriptions that start with “Constructed-” indicate variables that were created by the FFCWS, not the current authors. PCG= primary caregiver; SCG= secondary caregiver.

Supplemental Table 2. Independent and Interactive Effects (with standard errors) of Adverse Childhood Experiences (ACEs) and Household Income on Adolescent Depression Symptoms.

	Depression Symptoms (n=2,348)					
	**Model 1**	**Model 2**	**Model 3**	**Model 4**	**Model 5**	**Model 6**
**Household Income**						
<50% FPL	(ref)				(ref)	(ref)
50-99% FPL	-0.02 (0.04)				-0.01 (0.04)	-0.06 (0.10)
100-199% FPL	-0.05 (0.04)				-0.05 (0.04)	-0.00 (0.09)
200-299% FPL	-0.06 (0.04)				-0.04 (0.04)	-0.07 (0.10)
≥300% FPL	**-0.11 (0.04)****				**-0.09 (0.04)***	-0.00 (0.09)
**ACE Indices**						
Maltreatment		0.02 (0.02)		0.01 (0.02)	0.01 (0.02)	0.00 (0.04)
Parent Challenges			**0.06 (0.01)*****	**0.06 (0.01)*****	**0.06 (0.01)*****	**0.07 (0.03)***
**Interactions**						
Maltreatment (CM) x Household Income						
CM x 0-49% FPL						(ref)
CM x 50-99% FPL						0.02 (0.05)
CM x 100-199% FPL						-0.01 (0.05)
CM x 200-299% FPL						0.04 (0.05)
CM x ≥300% FPL						0.01 (0.05)
Parent Challenges (PC) x Household Income						
PC x 0-49% FPL						(ref)
PC x 50-99% FPL						0.01 (0.04)
PC x 100-199% FPL						-0.02 (0.04)
PC x 200-299% FPL						-0.03 (0.04)
PC x ≥300% FPL						-0.01 (0.04)
Youth age	0.00 (0.00)	0.02 (0.00)	0.00 (0.00)	0.00 (0.00)	0.00 (0.00)	0.00 (0.00)
Youth race/ethnicity						

White, non-Hispanic	(ref)	(ref)	(ref)	(ref)	(ref)	(ref)
Black, non-Hispanic	-0.03 (0.04)	-0.00 (0.04)	-0.01 (0.04)	-0.01 (0.04)	-0.03 (0.04)	-0.03 (0.04)
Hispanic/Latino	0.02 (0.04)	0.05 (0.04)	0.04 (0.04)	0.04 (0.04)	0.02 (0.04)	0.02 (0.04)
Multiracial/other	0.07 (0.06)	0.08 (0.05)	0.07 (0.05)	0.07 (0.05)	0.06 (0.05)	0.06 (0.06)
Youth sex						
Male	(ref)	(ref)	(ref)	(ref)	(ref)	(ref)
Female	**0.12 (0.02)***	**0.12 (0.02)***	**0.13 (0.02)***	**0.13 (0.02)***	**0.13 (0.02)***	**0.13 (0.02)***
Mom age	-0.00 (0.00)	-0.00 (0.00)	0.00 (0.00)	0.00 (0.00)	0.00 (0.00)	0.00 (0.00)
Mom Nativity						
U.S. Born	(ref)	(ref)	(ref)	(ref)	(ref)	(ref)
Not U.S. Born	0.03 (0.04)	0.03 (0.04)	0.04 (0.04)	0.04 (0.04)	0.04 (0.04)	0.05 (0.04)
Parent Relationship Status						
Married	(ref)	(ref)	(ref)	(ref)	(ref)	(ref)
Cohabiting	0.05 (0.04)	**0.07 (0.04)***	0.06 (0.04)	0.05 (0.04)	0.03 (0.04)	0.04 (0.04)
Neither	0.04 (0.04)	**0.08 (0.04)***	0.07 (0.04)†	0.07 (0.04)†	0.04 (0.04)	0.04 (0.04)
Income Imputed						
Not Imputed	(ref)	(ref)	(ref)	(ref)	(ref)	(ref)
Imputed	0.02 (0.04)	0.02 (0.04)	0.01 (0.04)	0.01 (0.04)	0.01 (0.04)	0.02 (0.04)

Note: Abbreviations are FPL, federal poverty level; ACEs, Adverse Childhood Experiences; CM, child maltreatment; PC, parent challenges. Bolded values indicate statistical significance at p≤0.05; *p≤0.05, **p<0.01, ***p<0.001, † p<0.10. Youth sex and maternal characteristics were assessed at the time of the child's birth. Youth age (in months) and race/ethnicity were assessed at the age 15 survey.

Supplemental Table 3. Independent and Interactive Effects (with standard errors) of Adverse Childhood Experiences (ACEs) and Household Income on Adolescent Anxiety Symptoms

	**Anxiety Symptoms (n=2,386)**					
	**Model 1**	**Model 2**	**Model 3**	**Model 4**	**Model 5**	**Model 6**
**Household Income**						
<50% FPL	(ref)				(ref)	(ref)
50-99% FPL	-0.02 (0.05)				-0.01 (0.05)	0.00 (0.10)
100-199% FPL	-0.07 (0.04)†				-0.06 (0.04)	0.04 (0.09)
200-299% FPL	-0.08 (0.05)				-0.06 (0.05)	0.03 (0.11)
≥300% FPL	-0.09 (0.05)†				-0.06 (0.05)	-0.03 (0.10)
**ACE Indices**						
Maltreatment		0.02 (0.02)		0.01 (0.02)	0.01 (0.02)	-0.01 (0.04)
Parent Challenges			**0.06 (0.01)*** **	**0.06 (0.01)*** **	**0.06 (0.01)*** **	**0.12 (0.03)*** **
**Interactions**						
Maltreatment (CM) x Household Income						
CM x 0-49% FPL						(ref)
CM x 50-99% FPL						0.06 (0.06)
CM x 100-199% FPL						0.00 (0.05)
CM x 200-299% FPL						0.03 (0.06)
CM x ≥300% FPL						0.02 (0.06)
Parent Challenges (PC) x Household Income						
PC x 0-49% FPL						(ref)
PC x 50-99% FPL						-0.08 (0.04) †
PC x 100-199% FPL						**-0.09 (0.04)***
PC x 200-299% FPL						**-0.12 (0.05)***
PC x ≥300% FPL						-0.05 (0.04)
Youth age	-0.00 (0.00)	-0.00 (0.00)	-0.00 (0.00)	-0.00 (0.00)	-0.00 (0.00)	-0.00 (0.00)
Youth race/ethnicity						

White, non-Hispanic	(ref)	(ref)	(ref)	(ref)	(ref)	(ref)
Black, non-Hispanic	**-0.09 (0.04)***	**-0.08 (0.04)***	**-0.08 (0.04)***	**-0.08 (0.04)***	**-0.10 (0.04)***	**-0.10 (0.04)***
Hispanic/Latino	0.00 (0.05)	0.02 (0.05)	0.02 (0.05)	0.02 (0.05)	0.01 (0.05)	0.01 (0.05)
Multiracial/other	-0.04 (0.06)	-0.03 (0.06)	-0.04 (0.06)	-0.04 (0.06)	-0.04 (0.06)	-0.04 (0.06)
Youth sex						
Male	(ref)	(ref)	(ref)	(ref)	(ref)	(ref)
Female	**0.07 (0.03)****	**0.07 (0.03)****	**0.08 (0.03)****	**0.08 (0.03)****	**0.08 (0.03)****	**0.08 (0.03)****
Mom age	-0.00 (0.00)	-0.00 (0.00)	-0.00 (0.05)	-0.00 (0.00)	-0.00 (0.00)	-0.00 (0.00)
Mom Nativity						
U.S. Born	(ref)	(ref)	(ref)	(ref)	(ref)	(ref)
Not U.S. Born	0.01 (0.05)	0.01 (0.05)	0.02 (0.05)	0.02 (0.05)	0.02 (0.05)	0.02 (0.05)
Parent Relationship Status						
Married	(ref)	(ref)	(ref)	(ref)	(ref)	(ref)
Cohabiting	**0.08 (0.04)***	**0.09 (0.04)***	**0.08 (0.04)***	**0.08 (0.04)***	0.07 (0.04)	0.07 (0.04)
Neither	0.6 (0.04)	**0.08 (0.04)***	0.07 (0.04)†	0.07 (0.04)†	0.05 (0.04)	0.05 (0.04)
Income Imputed						
Not Imputed	(ref)	(ref)	(ref)	(ref)	(ref)	(ref)
Imputed	0.01 (0.04)	0.00 (0.04)	0.00 (0.04)	-0.00 (0.04)	0.01 (0.04)	0.01 (0.04)

Note: Abbreviations are FPL, federal poverty level; ACEs, Adverse Childhood Experiences; CM, child maltreatment; PC, parent challenges. Bolded values indicate statistical significance at $p \leq 0.05$; *$p \leq 0.05$, **$p<0.01$, ***$p<0.001$, † $p<0.10$. Youth sex and maternal characteristics were assessed at the time of the child's birth. Youth age (in months) and race/ethnicity were assessed at the age 15 survey.

Supplemental Table 4. Independent and Interactive Effects (with standard errors) of Adverse Childhood Experiences (ACEs) and Household Income on Adolescent Depression (top) and Anxiety Symptoms (bottom) using lower response thresholds for the Child Maltreatment indicators.

**Depression Symptoms (n=2,348)**						
	**Model 1**	**Model 2**	**Model 3**	**Model 4**	**Model 5**	**Model 6**
**Household Income**						
<50% FPL	(ref)				(ref)	(ref)
50-99% FPL	-0.02 (0.04)				-0.01 (0.04)	0.09 (0.11)
100-199% FPL	-0.05 (0.04)				-0.05 (0.04)	0.14 (0.10)
200-299% FPL	-0.06 (0.04)				-0.04 (0.04)	0.02 (0.11)
≥300% FPL	**-0.11 (0.04)***				**-0.09 (0.04)***	-0.04 (0.10)
**ACE Indices**						
Maltreatment		**0.04 (0.02)***		0.03 (0.02)	0.03 (0.02)†	**0.09 (0.04)***
Parent Challenges			**0.06 (0.01)*****	**0.06 (0.01)*****	**0.05 (0.01)*****	**0.06 (0.03)***
**Interactions**						
Maltreatment (CM) x Household Income						
CM x 0-49% FPL						(ref)
CM x 50-99% FPL						-0.08 (0.06)
CM x 100-199% FPL						**-0.11 (0.05)***
CM x 200-299% FPL						-0.03 (0.06)
CM x ≥300% FPL						-0.03 (0.06)
Parent Challenges (PC) x Household Income						
PC x 0-49% FPL						(ref)
PC x 50-99% FPL						0.02 (0.04)
PC x 100-199% FPL						-0.01 (0.04)
PC x 200-299% FPL						-0.02 (0.04)
PC x ≥300% FPL						-0.01 (0.04)
**Anxiety Symptoms (n=2,386)**						

**Household Income**						
<50% FPL	(ref)				(ref)	(ref)
50-99% FPL	-0.02 (0.05)				-0.01 (0.05)	0.10 (0.12)
100-199% FPL	-0.07 (0.04)†				-0.07 (0.04)	0.14 (0.11)
200-299% FPL	-0.08 (0.05)				-0.06 (0.05)	0.09 (0.12)
≥300% FPL	-0.09 (0.05)†				-0.06 (0.05)	-0.05 (0.11)
**ACE Indices**						
Maltreatment		0.02 (0.02)		0.01 (0.02)	0.02 (0.02)	0.03 (0.05)
Parent Challenges			**0.06 (0.01)*** **	**0.06 (0.01)*** **	**0.06 (0.01)*** **	**0.12 (0.03)*** **
**Interactions**						
Maltreatment (CM) x Household Income						
CM x 0-49% FPL						(ref)
CM x 50-99% FPL						0.01 (0.07)
CM x 100-199% FPL						0.07 (0.06)
CM x 200-299% FPL						0.02 (0.07)
CM x ≥300% FPL						0.03 (0.06)
Parent Challenges (PC) x Household Income						
PC x 0-49% FPL						(ref)
PC x 50-99% FPL						-0.07 (0.04) †
PC x 100-199% FPL						**-0.08 (0.04)***
PC x 200-299% FPL						**-0.11 (0.05)***
PC x ≥300% FPL						-0.05 (0.04)

Note: Abbreviations are FPL, federal poverty level; ACEs, Adverse Childhood Experiences; CM, child maltreatment; PC, parent challenges. Bolded values indicate statistical significance at p≤0.05; *p≤0.05, **p<0.01, ***p<0.001, † p<0.10. Demographic covariates were included in all models but are not shown here.

Supplemental Table 5. Marginal effects (or dy/dx, indicating the change in outcome associated with a minute change in exposure) and standard errors for increasing Child Maltreatment (using lower thresholds to indicate individual CM variables) & Parent Challenges by Income Category

Income	Depression		Anxiety	
	**CM**	**PC**	**CM**	**PC**
<50% FPL	0.09 (0.04)*	0.06 (0.03)*	0.03 (0.05)	0.12 (0.03)***
50-99% FPL	0.01 (0.04)	0.08 (0.03)*	0.02 (0.05)	0.05 (0.03)
100-199% FPL	-0.03 (0.03)	0.05 (0.02)†	-0.03 (0.04)	0.04 (0.03)
200-299% FPL	0.06 (0.04)	0.04 (0.03)	0.02 (0.05)	0.01 (0.04)
≥300% FPL	0.05 (0.04)	0.05 (0.03)†	0.07 (0.04)	0.07 (0.03)*

Note: ***p<0.001, **p<0.01, *p<0.5, †p<0.10. Abbreviations: CM, child maltreatment index; PC, parent challenges index; FPL, federal poverty level

Supplemental Table 6. Independent and Interactive Effects (with standard errors) of Adverse Childhood Experiences (ACEs) and Household Income on Adolescent Depression (top) and Anxiety (bottom) using the entire baseline sample and full information maximum likelihood (FIML) estimation to address missingness.

**Depressive Symptoms (n=4,898)**						
	**Model 1**	**Model 2**	**Model 3**	**Model 4**	**Model 5**	**Model 6**
**Household Income**						
<50% FPL	(ref)				(ref)	(ref)
50-99% FPL	-0.01 (0.03)				-0.00 (0.03)	-0.00 (0.08)
100-199% FPL	-0.06 (0.03)				-0.06 (0.03)	-0.03 (0.08)
200-299% FPL	-0.05 (0.04)				-0.04 (0.04)	-0.06 (0.09)
≥300% FPL	**-0.13 (0.04)*** **				**-0.11 (0.04)** **	-0.13 (0.08)
**ACE Indices**						
Maltreatment		0.02 (0.02)		0.01 (0.02)	0.01 (0.02)	0.02 (0.03)
Parent Challenges			**0.06 (0.01)*** **	**0.06 (0.01)*** **	**0.05 (0.01)*** **	**0.07 (0.03)* **
**Interactions**						
Maltreatment (CM) x Household Income						
CM x 0-49% FPL						(ref)
CM x 50-99% FPL						0.01 (0.05)
CM x 100-199% FPL						-0.01 (0.05)
CM x 200-299% FPL						0.05 (0.05)
CM x ≥300% FPL						0.01 (0.05)
Parent Challenges (PC) x Household Income						
PC x 0-49% FPL						(ref)
PC x 50-99% FPL						-0.01 (0.03)
PC x 100-199% FPL						-0.01 (0.03)
PC x 200-299% FPL						-0.06 (0.04)
PC x ≥300% FPL						0.00 (0.03)
**Anxiety Symptoms (n=4,898)**						
**Household Income**						

<50% FPL	(ref)				(ref)	(ref)
50-99% FPL	-0.01 (0.04)				-0.01 (0.04)	0.00 (0.09)
100-199% FPL	**-0.07 (0.03)***				-0.07 (0.03)	0.00 (0.08)
200-299% FPL	-0.05 (0.04)				-0.04 (0.04)	0.05 (0.09)
≥300% FPL	**-0.11 (0.04)****				**-0.09 (0.04)***	-0.09 (0.09)
**ACE Indices**						
Maltreatment		0.02 (0.02)		0.01 (0.02)	0.01 (0.02)	0.02 (0.04)
Parent Challenges			**0.05 (0.01)*****	**0.05 (0.01)*****	**0.05 (0.01)*****	**0.09 (0.03)****
**Interactions**						
Maltreatment (CM) x Household Income						
CM x 0-49% FPL						(ref)
CM x 50-99% FPL						0.06 (0.05)
CM x 100-199% FPL						0.01 (0.05)
CM x 200-299% FPL						0.03 (0.06)
CM x ≥300% FPL						0.03 (0.05)
Parent Challenges (PC) x Household Income						
PC x 0-49% FPL						(ref)
PC x 50-99% FPL						**-0.08 (0.04)***
PC x 100-199% FPL						**-0.07 (0.03)***
PC x 200-299% FPL						**-0.13 (0.04)****
PC x ≥300% FPL						-0.02 (0.04)

Note: Abbreviations are FPL, federal poverty level; ACEs, Adverse Childhood Experiences; CM, child maltreatment; PC, parent challenges.
Bolded values with indicate statistical significance at $p<0.05$; ***$p<0.001$, **$p<0.01$, *$p<0.05$, † $p<0.10$.
Demographic covariates were included in all models but are not shown here; full tables can be found in the Supplemental Materials (Supplemental Tables 1 & 2).
There was a significant difference in the effect sizes of household income ≥300% FPL versus Parent Challenges in relation to depression symptoms in postestimation comparisons.
